# Initial Validation and Findings From the Willing/Ready Subscale of the Church Addiction Response Scale

**DOI:** 10.3389/fpsyg.2021.733913

**Published:** 2021-10-18

**Authors:** Andrea D. Clements, Natalie Cyphers, Deborah L. Whittaker, Brett McCarty

**Affiliations:** ^1^Department of Psychology, East Tennessee State University, Johnson City, TN, United States; ^2^Uplift Appalachia, Johnson City, TN, United States; ^3^East Tennessee State University Ballad Health Strong BRAIN Institute, Johnson City, TN, United States; ^4^Division of Nursing, DeSales University, Center Valley, PA, United States; ^5^Department of Population Health Sciences, Duke University School of Medicine, Durham, NC, United States; ^6^Duke Divinity School, Duke University, Durham, NC, United States

**Keywords:** church readiness, religion and health research, addiction, measurement, church mobilization, substance use disorder

## Abstract

Addiction has been a global health crisis over recent decades and worsened substantially during COVID-19 lockdowns. We report on the development, validation, and findings from an instrument developed to assess the readiness of churches in the Appalachian Highlands to address addiction. The Church Addiction Response Scale (CARS) is a 41-item, three section measure assessing “What are your views about addiction?” (14 items), “What are your views about interacting with people who are addicted to drugs?” (11 items), and “What do you think the church’s role is in addressing addiction?” (16 items). The CARS was found to be unidimensional with strong internal consistency and initial evidence of construct validity was positive. Most respondents reported willingness to assist people living with addiction, but many reported that they felt underprepared, thus were not ready. Areas of preparation were largely those that could be addressed through training, such as understanding the physiology and psychology of addiction, available treatment options, and how to avoid doing harm. Thus, with adequate training, the likelihood of equipping a church-based workforce to provide support for people living with addiction seems attainable.

## Introduction

An alarm is being sounded across the nation about the ravages of addiction to substances and increasingly communities of faith are the target of that call (Davis, 2018; Stetzer, 2018; Worthington, 2018). According to the Centers for Disease Control and Prevention (Centers for Disease Control and Prevention [CDC], 2017) the rate of deaths from overdose in the United States (U.S.) was five times higher in 2016 than in 1999 and has increased dramatically during the time of COVID-19 lockdowns. Many states report overdose death rate increases of over 30% compared to 2019, with the annual overdose death rate already exceeding any previous year. In the 12 months ending November 2020, which included the first 6 months of the COVID-19 pandemic, the number of overdose deaths was over 93,000, exceeding the highest previous 12-month period by 20,000 (Centers for Disease Control and Prevention [CDC], 2021). In the counties in which the current study was conducted, the overdose death rate ranged from 30 to 50/100 K, which exceeds the national rate (28.7/100 K; National Opinion Research Center [NORC], n.d.). Tennessee ranks 3rd in the nation for opioid prescribing with a regional rate of 112 per 100 people (Brantley, 2019). Those numbers are decreasing for opioids prescribed for pain, however, opioids prescribed for addiction treatment have risen steadily, are often diverted, and are responsible for over 80% of neonatal abstinence syndrome (NAS), also known as neonatal opioid withdrawal syndrome (NOWS), in infants born in this region (Olsen, 2020). Problematic use of substances is not limited to opioids, or to the United States, however. Just prior to the pandemic, the United Nations Office on Drugs and Crime (United Nations Office on Drugs and Crime [UNODC], 2020) report that over 35 million people worldwide are classified as having drug use disorders, up more than 30% since 2009. This report includes monitoring of opioids, cocaine, cannabis, amphetamine-type stimulants such a methamphetamine, and new psychoactive substances (NPS).

The cost of this epidemic extends beyond multi-billion-dollar economic costs to deeply personal costs to millions of Americans, their families, and communities (U.S. Department of Health and Human Services, n.d.). There is a growing recognition that faith-based organizations can play constructive roles in response to public health issues (Idler et al., 2019). Communities and churches are being challenged to respond constructively to the opioid crisis, but are they responding? The purpose of this research is to describe the readiness of churches in the Appalachian Highlands to address not only the opioid crisis, but problematic use of any substance.

Research has shown that intensive behavioral treatment (close monitoring, accountability) is quite effective in reducing use of many addictive substances (Substance Abuse and Mental Health Services Administration [SAMHSA], 2018). Even with this understanding, the staffing needs are too great to offer the level of needed support through paid personnel. Having a large volunteer workforce, such as equipped churchgoers, can help to meet this need (Stahler et al., 2007; Kerley et al., 2010; Stacer and Roberts, 2018; Glazier, 2020). The faith community can offer behavioral support (building coping skills, changing thought patterns, accountability) and social support to reduce the desire to self-medicate (dealing with past adversity through supportive relationships). Pillion (2017), a State House delegate from nearby Dickenson County, Virginia, stated, *“*…*churches and ministries have a unique role in this important conversation and efforts to assist individuals and families in need. The power in being part of a loving community that helps take away the stigma of addiction cannot be underestimated. People are hungry for a place to feel welcome, supported, and loved*.”

Within the metro area in which data were collected, there were just over 500,000 people, of which over 266,000 identified as religious, with the vast majority identifying as Evangelical Christians (The Association of Religion Data Archives [ARDA], 2010). The Pew Research Center (2019) has found that nationwide approximately 54% of individuals identifying as religious are active in church, so if that proportion were applied to this area, over 144,000 would be predicted to attend a church of some kind. Even a small proportion of this could be a beneficial workforce to care for people living with problematic substance use and its sequelae. But are these potential volunteers ready and willing?

Church-based health promoting programs have repeatedly been shown to be successful (e.g., DeHaven et al., 2004; Newlin et al., 2012; Baruth et al., 2013). For example, Epstein et al. (2007, 2009) demonstrated positive outcomes in a randomized controlled trial of substance abuse prevention educational program for school age children, however, they reported the churches’ response to participation in the study was disappointing. Such programs can only be helpful to the extent that churches implement them.

Stigma toward substance users is one potential explanation for low church engagement. In the general population, not churches specifically, the tendency to stigmatize differs by substance (Brown, 2015) and is greater toward those who are seen as resistant to changing their drug use behavior or who display low levels of willpower (Witte et al., 2019). Previous research has pointed out that congregational readiness to address addiction is predicted by experience with addictions, attitudes toward learning more about addiction and recovery, perceptions of being able to provide a supportive environment, and perceived willingness and ability to engage with someone living with addiction (Travis et al., 2012). Travis et al. (2012) noted significant readiness deficiencies within churches. Before implementing programs seeking to address the readiness deficiencies noted by Travis et al. (2012) information is needed regarding the specific readiness needs of churches.

A federal planning grant funded a regional effort to mobilize the faith community in the Appalachian Highlands to address addiction. One of the funding requirements was to conduct a needs assessment. A survey, the *Church Addiction Response Scale* (CARS), was developed as part of this needs assessment and it and its initial findings are the focus of the remainder of this article. The CARS is a self-report survey assessing three content domains, (1) *views about addiction*, (2) *views about interacting with people who are addicted to substances*, and (3) *views about the church*’*s role in addressing addiction*. We are reporting on preliminary validation efforts and initial findings for the CARS, focusing on churchgoer *willingness* and *readiness* to support people living with addictions or other problematic substance use.

## Materials and Methods

### Participants

Ethical review and approval was not required for this study as determined by the Institutional Review Board at East Tennessee State University. Written informed consent from the participants was not required to participate in this study in accordance with national legislation and institutional requirements. The 288 respondents (age 18–78 years; mean = 32.75, *SD* = 17.49; 194 female) who indicated they were involved in a church served as the sample for this study. Paper copies of the CARS were collected from participants (*n* = 16) at area interest meetings held by a non-profit organization that is seeking to mobilize the faith community to address addiction. An online version was available to undergraduate college students at a local university (*n* = 272).

### Measures

#### The Church Addiction Response Scale

The CARS is a 41-item, three section measure assessing “What are your views about addiction?” (14 items), “What are your views about interacting with people who are addicted to drugs?” (11 items), and “What do you think the church’s role is in addressing addiction?” (16 items) (see Table 1). All items are rated on a 5-point Likert scale (1 = *strongly agree* to 5 = *strongly disagree)*. Participants were given the following instructions prior to survey completion:

**TABLE 1:** Church Addiction Response Scale (1 = strongly agree to 5 = strongly disagree) overall means with group differences between online vs. in-person administration noted.

**Item**	**Mean**	**SD**
**Section 1. Views of addiction**		

God CAN take away someone’s addiction.	1.93**	1.22
If someone repents and prays honestly, God WILL ALWAYS take away their addiction.	3.19	1.40
Addiction is genetic/physical, so treatment can’t really help.	4.17	1.05
Someone who is addicted to substances could stop if they really wanted to.	3.14**	1.35
Past experiences can drive someone to misuse substances.	1.34	0.59
I would be embarrassed to tell someone that I or a loved one was struggling with addiction.	2.82	1.40
Addiction is a medical issue so it should be treated medically.	2.26*	1.13
Addiction treatment isn’t very effective.	3.62**	1.04
People who are addicted to substances should stop using them completely and not take substitutes.	2.97	1.28
People should be allowed to die of overdoses.	4.38***	1.11
People who are addicted to substances are valuable.	1.36***	0.80
People need to muster up enough willpower not to use substances.	2.80***	1.26
Money spent helping people who are trying to overcome addiction is money well spent.	1.70	0.88
Addiction should be handled by professionals.	1.67**	0.88

**Section 2. Views of interacting with people addicted to drugs**		

*I want to model to my loved ones that it is good to help someone who is addicted to substances.*	1.45	0.71
*I might do something wrong or make things worse if I try to help someone who is addicted to substances.*	3.22	1.12
*I’m afraid I might be injured or catch a disease if I try to help people who are addicted to substances.*	4.06	1.11
*It’s too hard emotionally to help people who are addicted to substances.*	3.38	1.17
*It’s too expensive to try to help people who are addicted to substances.*	3.74***	1.16
***I want to help someone who is trying to overcome addiction any way I can.***	1.69***	0.80
*I don’t want to be an enabler so I shouldn’t help people who are addicted to substances.*	3.88*	1.13
*God judges people who are addicted to substances, so I shouldn’t be soft on them.*	4.59	0.83
*God loves people who are addicted to substances, so I should too.*	1.19	0.64
*If someone had overdosed, I would be willing to administer naloxone (Narcan) to try to save them if I was trained.*	1.32	0.71
*If someone I knew was addicted to substances, I would stay away from them.*	3.97***	1.07
*All people are struggling with something, so I should be able to empathize with people who are addicted to substances.*	1.55***	0.79

**Section 3. Church role in addressing addiction**		

*People in our church are willing to learn how to help people who are addicted to substances.*	1.99	1.03
*The church doesn’t know how to deal with addiction and might do something wrong or make things worse.*	3.39	1.16
*Our church is too poor to be able to do anything worthwhile to help people who are addicted to substances.*	4.27	0.99
*The church should be a place for someone who is addicted to substances to seek help.*	1.32	0.67
*People who are addicted to substances might damage the church (break things, steal things, get things dirty).*	3.57	1.16
*If people who are addicted to substances come into our church or religious services, it will make people nervous.*	3.25	1.18
*Since it’s the sick who need a physician, we should help people who are addicted to substances.*	1.73**	0.91
*Church funds should not be wasted on helping people who are addicted to substances.*	4.42	0.79
*If a church helps people who are addicted to substances, they will become dependent and keep wanting things.*	3.69*	1.02
*Churches should welcome people who are addicted to substances as they would anyone else.*	1.21	0.56
***Our church is already doing things to help people who are addicted to substances.***	2.35*	1.13
*I would fear for my family’s safety if people who are addicted to substances came to church.*	4.21	1.07
*If we allow people who are addicted to substances to be a part of our church, we will be perceived as being soft on sin.*	4.48	0.90
*If people who are addicted to substances go to a church, they may get more people in the church addicted.*	4.45	0.97
*Our church is too busy with other activities to minister to people who are addicted to substances.*	4.42	0.92

Italicized items were included in the *Willing/Ready Subscale*. Bold items were used as proxy for convergent validity investigation. Asterisks indicate questions on which groups differed significantly. All differences indicated that the paper group was more knowledgeable and more ready/willing (*p < 0.05, **p < 0.01, ***p < 0.001).

*This survey asks questions about your opinions on addiction and substance misuse. For this survey substance misuse is defined as repeated use of drugs or alcohol to feel good, reduce stress, and/or change or avoid reality. Substances include but are not limited to the following: alcohol, illegal substances and/or misuse of prescription drugs including use of prescription drugs in ways other than they were prescribed or use of someone else*’*s prescription medication.*

The first section assessed the individual’s views of addiction, such as whether they believe it is a medical issue, whether they believe people could stop using drugs if they wanted to, whether treatment is believed to be effective, and other general views of addiction. The remaining two sections assessed aspects of willingness and readiness. The second section assessed the individual, such as whether someone would be willing to help, or whether they would be afraid. The third section assessed the person’s perception of the church as ready or willing, such as asking whether church funds should be spent on addressing addiction. The latter two scales were combined into a 27-item *Willing/Ready Subscale* (mean = 110.89, *SD* = 12.79) that is being validated as a measure of church willingness/readiness to address addiction.

### Statistical Analysis Plan

Exploratory factor analysis (EFA) was conducted to determine whether factors were present. Items that were negatively related to the full measure were reverse scored so that lower item and total scores would indicate more willingness/readiness. In order to assess initial convergent validity with measures of both willingness and readiness to address addiction, the total of all items (except the item with which it was being correlated) was correlated with each of two items, one indicating willingness and one indicating readiness. The item that said, “I want to help someone who is trying to overcome addiction any way I can,” was used as a proxy for willingness. The item that said, “Our church is already doing things to help people who are addicted to drugs” was used as a proxy for church readiness. Descriptive statistics were calculated for total score on the *Willing/Ready Subscale*. Finally, independent *t*-tests were run on all items comparing means for the group.

## Results

### Initial Church Addiction Response Scale Validation

Exploratory factor analysis indicated that a one-factor solution was most parsimonious. Ten items that were negatively related to the full scale during EFA were reverse scored so that on all items and the total score, lower scores indicated greater willingness or readiness. Alternatively, high scores could be interpreted as hesitancy to enter this work. Internal consistency of the measure was high (α = 0.88) and was not significantly changed with removal of any item. To investigate convergent validity with measures of both willingness and readiness to address addiction, the total of all items except the item with which it was being correlated was correlated with each proxy item. The item that said, “I want to help someone who is trying to overcome addiction any way I can,” indicating willingness, was strongly positively correlated with the total of the remaining items (*r* = 0.50, *p* < 0.001). The item that said, “Our church is already doing things to help people who are addicted to drugs,” indicating readiness, was also strongly positively correlated with the total of the remaining items (*r* = 0.42, *p* < 0.001). An item from the first section of the CARS was used as an additional indicator of convergent validity. The total on the *Willing/Ready Subscale* was strongly positively correlated (*r* = 0.53, *p* < 0.001) with the item that stated, “Money spent helping people who are trying to overcome addiction is money well spent. Taken together, there is initial support for the *Willing/Ready Subscale* from the CARS to be used to predict willingness and readiness of churchgoers to address addiction.

### Initial Church Addiction Response Scale Willing/Ready Subscale Findings

Overall, most (>88%) respondents reported they would help someone living with addiction anyway they can, and 71.6% reported they thought the people in their church would be willing to learn to help. Other positive findings were that a vast majority (88%) thought that money spent on helping people with addiction is money well spent, and 90.5% would be willing to administer Narcan if trained. Additionally, almost everyone (97.1%) understood that past experiences can contribute to addiction. However, more than a quarter (27.2%) felt their church does not know what to do and over 30% felt that they might do something wrong.

Some areas indicating a need for training were identified with this survey as well. There was variation in what respondents believe about addiction, with some believing it is a medical issue that should only be handled medically (68.1%), some believing substitution medications should never be used (37.1%), and a small percentage believing treatment is ineffective (17.6%). Item means and standard deviations are included in Table 1. Also, 51.8% thought that people just need to “muster up enough willpower to stop” using substances, and 43.6% believe people can stop using substances if they really want to. Although willing to help for the most part, people did express concern about people living with addiction getting things dirty (25.5%), making people nervous (36.0%), fearing for their family’s safety (11.2%), worrying that additional church members will become addicted (6.1%), and being concerned that they might be personally injured (15.9%).

Although there were large differences in group size (16 in-person, 272 online), we investigated group differences between the in-person group who attended interest meetings about church mobilization to address addiction and the online, younger, primarily college student group, controlling for unequal variances when necessary. Significant differences were found for several items (*p*s ranged from <0.001 to 0.013), and where differences were found, the in-person responses were universally more favorable toward helping people living with addiction (e.g., addicted people are valuable, I want to help, it’s [not] too expensive to help, people should [not] be allowed to die of overdoses; see Table 1). The online group was more likely to view addiction as a medical issue [*t*(286) = 2.50, *p* = 0.013] but was more likely than the paper group to believe that treatment was ineffective [*t*(286) = 2.75, *p* = 0.005].

## Discussion

Initial validation of the *Willing/Ready Subscale* of the *Church Addiction Response Scale* is quite promising. Further validation through establishing convergent and discriminant validity and test-retest reliability are warranted, but in this initial validation, tested psychometric properties (e.g., internal consistency, validity) were found to be well-supported and respondents identified barriers to readiness that can be practically overcome.

It would be difficult to overcome barriers related to values, for example, if respondents had said they were not willing to help or did not find people living with addiction worthy of help. However, most of the barriers to readiness revealed by this instrument can be overcome through training regarding the science of addiction including likely origins of addiction and treatment options, as well as interaction with people who are addicted to substances. Both instilling an understanding about origins of addiction such as having a history of adverse childhood experiences (ACEs) (Felitti et al., 1998) and interacting with people who live with addictions both have been shown to reduce stigma at least sometimes (Corrigan and Nieweglowski, 2019; Sattler et al., 2021).

In the Appalachian Highlands, churches are plentiful, and a large proportion of the population attends church. Within these church walls is a potentially large volunteer workforce who could be mobilized to assist in addressing the problem of problematic substance use. According to this study, there seems to be strong support for churches being an appropriate place for addressing substance use. For the most part, respondents seemed to value the use of church funds and time to address addiction. Areas of mixed response primarily fell in four categories: (1) being unsure about the origins of and physiological aspects of addiction, (2) personal discomfort about danger, damage, and nervousness about interacting with people who differ (i.e., stigma), (3) lack of knowledge about how addiction should be treated, and (4) differing degrees of understanding about how the tenets of the Christian faith support care for people living with problematic substance use. It was apparent that those who were already involved in such work or who attended gatherings to investigate this work were more ready and willing than the general (and younger) churchgoing population.

Areas of training that could address some of the areas of expressed need include: (1) physical processes related to development of, continuation of, and treatment of addiction, (2) psychological processes related to development of, continuation of, and treatment of addiction, (3) pharmacological and non-pharmacological treatment options for addiction, (4) risk factors predictive of developing problematic substance use, such as poverty and ACEs, (5) how tenets of the Christian faith relate to understanding and caring for individuals who are addicted to substances, (6) what challenges are faced by people who live with addiction, and (7) ways to assist with those challenges. In addition, the needs assessment noted the greatest workforce shortages/gaps in helping with substance use problems included emotional/social support for people living with addiction and lack of transportation and housing. Providing training in the identified areas of need could move churchgoers from being willing and/or ready to help with these problems of substance use to active engagement with people with problematic substance use, thus facilitating healing.

Providing assistance to churchgoers as churches develop programs that meet some of the expressed practical needs of community members living with addiction (e.g., transportation, housing, job readiness) will give churchgoers opportunities to enhance treatment and recovery access. Equipping churches will deepen the commitment of churchgoers to invest time and resources into addressing addiction and may help to reduce the stigma toward individuals with problematic substance use histories.

One exemplar of a successful approach to expanding the volunteer workforce to address substance use concerns has been pilot tested by a non-profit organization called Uplift Appalachia, the developers of this instrument (Clements and Clements, 2020). Their approach included equipping a number of churchgoers within the targeted communities who became intrinsically motivated to physically, emotionally, and spiritually support people who were addicted to substances. Uplift Appalachia helped members of the volunteer workforce and people living with problematic substance use to gain a better understanding of scientifically supported mechanisms of addiction and addiction treatment, and then provided programs to enhance support while meeting practical needs. Uplift Appalachia determined the biggest practical need of individuals living with addiction was transportation to access treatment. The shortage of treatment options in rural areas places barriers on patients who must travel farther to access medication assisted treatment (MAT) and, in some cases, have to rely on friends or family for transportation (Rosenblum et al., 2011; Pullen and Oser, 2014; Sigmon, 2014). Given the mountainous terrain, travel is often time-consuming and costly. Many who live with substance use issues have lost driving privileges and/or cannot afford a vehicle or fuel. Although some services are provided online because of COVID-19, limited access to technology, limited skill using that technology, and limited broadband access can prevent access. Volunteers trained by Uplift Appalachia are able to better support people living with addiction and help the people living with addiction better understand their illness. Pilot data from a small church-based volunteer rideshare program confirmed that riders maintained sobriety and employment during the period in which they were receiving free transportation. The riders also expressed feelings of connection after receiving regular rides from volunteer drivers. Drivers reported feeling helpful and feeling less stigma toward recovering substance users. A more sophisticated study to investigate effects of this model is warranted as the original study did not have a control group comparison. The enhanced understanding about addiction and its origins, the development of relationships, and the meeting of practical needs through transportation are hypothesized to reduce the demand for substances among individuals who are addicted to them. Additionally, as churches become motivated to be a workforce, they may be mobilized more quickly if they are provided implementable programs such as this. We believe implementation of a church-based transportation program can help to improve access to treatment and recovery for people who live with addiction while building supportive relationships, thus meeting two documented needs (access and support).

In conclusion, this instrument seems to be a helpful tool for gauging readiness and willingness of the faith community to support individuals with problematic substance use. To date, most of the barriers to action identified revolve around a need for further training about addiction, rather than a perception that the work is unimportant, or that the people are not valued. Pilot testing of a church-based rideshare program has shown that churchgoers can be trained and engaged in efforts to care for people living with addiction. Having prepared programming may facilitate engagement in this work.

## Data Availability Statement

The raw data supporting the conclusions of this article will be made available by the authors, without undue reservation.

## Ethics Statement

The studies involving human participants were reviewed and approved by the East Tennessee State University Institutional Review Board. Written informed consent for participation was not required for this study in accordance with the national legislation and the institutional requirements.

## Author Contributions

AC was PI of the funded project during which church readiness data were collected, outlined the article, conducting statistical analyses, and wrote much of the methods and results. NC contributed to choosing the direction of the article, and collaborated on validation efforts, conducted analyses, and contributed to the literature collection and writing. DW was instrumental in assessing clarity for a healthcare audience, edited wording, suggested needed literature, edited both body and references, and participated in brainstorming meetings throughout. BM was involved in the initial needs assessment discussion, has conducted parallel qualitative research in this same region, contributed to the literature collection and writing, and checked the accuracy of religious and cultural descriptions, both locally and generally. All authors were part of a research group who are focused on church mobilization to address addiction.

## Conflict of Interest

The authors declare that the research was conducted in the absence of any commercial or financial relationships that could be construed as a potential conflict of interest.

## Publisher’s Note

All claims expressed in this article are solely those of the authors and do not necessarily represent those of their affiliated organizations, or those of the publisher, the editors and the reviewers. Any product that may be evaluated in this article, or claim that may be made by its manufacturer, is not guaranteed or endorsed by the publisher.
